# Sexual Practices Among Residents Over 18 Years of Age in the Santa Rosa Sector of Baní, Dominican Republic: Risk Factors Associated With the Transmission of Sexually Transmitted Infections

**DOI:** 10.7759/cureus.88170

**Published:** 2025-07-17

**Authors:** Paola Rivera, Gabriela Pichardo, Geraldo Brito, Ariadna Somoza

**Affiliations:** 1 Department of Medicine, Instituto Tecnologico de Santo Domingo, Santo Domingo, DOM

**Keywords:** exposition, risk factors, sexual education, sexual practices, stis

## Abstract

Introduction: Sexually transmitted infections (STIs) constitute a diverse group of diseases that spread among individuals through sexual contact and nonsexual exposures, such as blood transfusions or contamination with organic fluids. These infections are caused by a variety of pathogens, including bacteria, viruses, protozoa, and parasites, among others.

Objectives: This study aimed to identify which gender most frequently engages in risky sexual behaviors, determine the age group most susceptible to contracting STIs within the study population, evaluate which level of education is associated with a higher likelihood of STI occurrence in the community, describe the most common individual and partner-related risk behaviors, assess whether participants had received any form of sexual education, and analyze how often condoms are used, with attention to differences according to sex.

Methodology: This is an observational, cross-sectional, prospective, and descriptive study with a population sample of 346 participants.

Results: The median age for first sexual intercourse was 17 years, the most common STI in the population was vaginal trichomonas infection, and in a history of sexual education, more people reported having received some type of sexual education. The most relevant risky sexual practices in people with a history of sexual education reported were the use of condoms, with 47% never using them, the number of sexual partners, and the type of these sexual relations.

Conclusions: Despite some sexual education, risky practices such as inconsistent condom use and multiple partners persist, highlighting the importance of personalized and reinforced educational programs to effectively address these behaviors.

## Introduction

Sexually transmitted infections (STIs) constitute a heterogeneous group of diseases transmitted from person to person through sexual contact, as well as through nonsexual means, such as blood transfusions or contamination with bodily fluids. These infections are caused by various pathogens, including bacteria, viruses, protozoa, and parasites, among others [1].

According to the World Health Organization (WHO), STIs have a significant impact on sexual and reproductive health worldwide. These diseases affect a large population, with more than one million new infections reported daily. In 2020, WHO reported 374 million new infections from four specific types of STIs: chlamydia (129 million), gonorrhea (82 million), syphilis (7.1 million), and trichomoniasis (156 million). Additionally, in 2016, more than 490 million people were living with genital herpes, and around 300 million women were infected with human papillomavirus (HPV), the leading cause of cervical cancer, as well as anal cancer in men who have sex with men. Approximately 296 million individuals globally are estimated to suffer from chronic hepatitis B [2].

Currently, more than 30 different bacteria, viruses, and parasites are known to be sexually transmitted through vaginal, anal, or oral sex. Of these, eight are the most prevalent STIs. Four are curable: syphilis, gonorrhea, chlamydia, and trichomoniasis. The other four are viral infections that currently have no cure: hepatitis B, herpes simplex virus, HIV, and HPV [2].

The impact of these infections extends beyond immediate consequences, as they can have serious long-term effects. For instance, STIs like herpes, gonorrhea, and syphilis are associated with an increased risk of acquiring HIV. Transmission of STIs from mother to child during pregnancy can result in severe complications, such as prenatal or neonatal death, prematurity, low birth weight, sepsis, neonatal conjunctivitis, and various congenital anomalies. Hepatitis B alone was responsible for an estimated 820,000 deaths in 2019, primarily due to complications like cirrhosis or hepatocellular carcinoma [2].

Additionally, new outbreaks of infections transmissible through sexual contact, such as monkeypox, infections from *Shigella sonnei* or *Neisseria meningitidis*, Ebola, and Zika, pose increasing challenges. There has also been a resurgence of neglected STIs like lymphogranuloma venereum, which underscores the growing difficulties in delivering adequate prevention and control services [2].

The Pan American Health Organization reports that new HIV infections in Latin America increased by 4.7% between 2010 and 2021, reaching approximately 110,000 cases in 2021. Meanwhile, the Caribbean saw a 28% reduction in new infections, dropping from 19,000 in 2010 to 14,000 in 2021 [3]. In the Dominican Republic, according to the most recent report from the National Council for HIV and AIDS in 2021, the HIV epidemic has not changed significantly. In 2009, the prevalence was 0.9%, and by 2021, it had decreased to 0.88%. There are major educational disparities: HIV prevalence is 54 times higher among uneducated women compared to those with higher education. Among women aged 23-24, the prevalence is 13 times higher than in those aged 15-17. Among men, the prevalence increases between the ages of 24 and 29, from 0.2% to 1.7%. The estimated total number of people living with HIV in 2021 was 75,793 [4].

Certain sexual behaviors significantly increase the likelihood of contracting an STI. These include having unprotected sex, having multiple sexual partners, and engaging in sexual activity under the influence of alcohol or drugs. Other risk factors vary by population and context, such as lack of sexual education, limited access to sexual health services, and failure to use barrier methods [5]. Several studies have identified similar risk factors in adolescents and young adults, particularly in vulnerable educational contexts, as previously observed in research conducted among high school students in Latin America [1].

The true extent of the STI problem is underestimated due to the nature of the diseases and flaws in reporting systems. The associated stigma and the fact that most STIs are asymptomatic or subclinical result in lower detection rates [6]. In the province where this study was conducted, there is no comprehensive data on current STI statistics, aside from the data gathered from the Hospital Our Lady of Regla. In 2022, the hospital reported 82 new HIV cases and 11 cases of syphilis in pregnant women. In the current year, 84 new HIV cases and six syphilis cases in pregnant women have been recorded. However, these figures likely underestimate the problem, as data for other STIs remain unavailable. Furthermore, they reveal a lack of routine testing practices, as diagnoses such as for syphilis are mostly identified during prenatal evaluations, leaving the broader sexually active population largely untested. This suggests a lack of awareness or habit of undergoing STI screening.

In this context, there is a clear need for a deeper and more comprehensive understanding of the implications and consequences of STIs, as well as the implementation of effective strategies for their prevention and control, to address this global public health challenge [2].

## Materials and methods

Study design

This study employed an observational, cross-sectional, prospective, and descriptive design. The research report was carried out in accordance with the Strengthening the Reporting of Observational Studies in Epidemiology guidelines.

Context

The study was conducted among permanent volunteer residents of the Santa Rosa Sector in the Municipality of Baní, Peravia Province, Dominican Republic, from November to December 2023. Data collection took place from December 3 to 13, 2023.

Participants

The study population was obtained from data provided by the primary care center located in Santa Rosa. In collaboration with a cartographer, this center carried out a division of the sector into three zones and a segmentation of the populations identified as Santa Rosa 1, Santa Rosa 2, and Santa Rosa 3. The investigation was conducted exclusively with residents of Santa Rosa 2 (see the Appendix) for convenience of location. In this area, 3,224 inhabitants were registered, of whom 2,088 were adults.

For data collection, volunteers were selected who met the following criteria: 1) individuals over 18 years old, 2) permanent residents of the Santa Rosa 2 neighborhood, Baní, Dominican Republic, 3) individuals who provided informed consent, and 4) individuals who spoke Spanish or Creole. Participants with cognitive disabilities were excluded.

To calculate the sample size, the finite population sample size formula was used, administered through the Epi Info application of the Centers for Disease Control and Prevention, Atlanta, GA, using a 95% confidence level, 5% margin of error, and a p value of 50%, resulting in a sample size of 324 participants (see the Appendix). A nonprobabilistic convenience sampling was conducted, applying the questionnaire to available and willing residents of Santa Rosa 2. Additionally, people who attended the primary care center and lived within the boundaries of the studied sector were surveyed. A total of 361 samples were collected, of which 16 were discarded due to being incomplete, resulting in a total of 345 questionnaires evaluated.

Variables and data collection tool

For data collection, a closed-ended form was developed (see the Appendix) adapted from the “Working instrument for the study of sexually transmitted diseases and HIV/AIDS in adolescents” developed by the team led by Cortés Alfaro et al. [7]. The adaptation was made in order to adjust the variables of interest specific to our study population, consisting of a questionnaire composed of 24 questions distributed into four sections: the first collects sociodemographic data, the second gathers behavioral data from both the participant and their partner, the third addresses personal history from both the individual and their current partner, and the fourth focuses on obtaining information about the participants’ history of sexual education.

In this questionnaire, variables were measured with both quantitative and qualitative approaches. Among the evaluated discrete quantitative variables were age and age of first sexual intercourse. Likewise, qualitative ordinal variables, such as educational level, were measured; dichotomous variables, such as gender; and qualitative nominal polytomous variables, covering aspects such as nationality, marital status, history of sexual education, high-risk sexual practices, and history of STIs.

This questionnaire was administered to users by collaborators in printed copies, available in Spanish and Creole, and delivered to participants according to their language preference. These were completed independently and confidentially by each participant. Subsequently, the questionnaires were entered into the Google Forms platform (Google LLC, Mountain View, CA), facilitating the calculation and management of the data.

Statistical methods and data management

To analyze the discrete quantitative variables of age and age of first sexual intercourse, a descriptive analysis was performed. The Shapiro-Wilk test (n > 50) was used through IBM Statistical Package for the Social Sciences, version 25 (IBM Corp., Armonk, NY) to measure the frequency and trend of the data. The results revealed a p value for the age variable of 1.992 × 10^-12^, which is less than α = 0.05. This indicates that the data do not follow a normal distribution. Similarly, for the variable age of first sexual intercourse, the p value was 8.305 × 10^-10^, also less than α = 0.05. Therefore, the median was used as the measure of central tendency and the interquartile range as the measure of dispersion. Variables such as gender, nationality, marital status, high-risk sexual practices, and history of STIs were evaluated based on the obtained frequencies. The results of this research were tabulated and graphically represented using Microsoft Excel 2022 (Microsoft Corporation, Redmond, WA).

Bias prevention

We anticipated the possible presence of biases commonly associated with self-reported data, including response bias, defined as the tendency of respondents to answer in a way that distorts reality, and social desirability bias. To minimize these effects, participants were assured of complete confidentiality when completing the questionnaires. The Hawthorne effect, which occurs when individuals modify their behavior due to awareness of being observed, was addressed by administering individual questionnaires rather than conducting group interviews. Recall bias, which can arise when participants fail to accurately remember past events, was mitigated by formulating specific and detailed questions rather than open-ended or general ones. This approach facilitated more precise recollections and contributed to the accuracy and quality of the collected data [8].

Ethical considerations

The criterion of confidentiality was supported by the use of informed consent available in Spanish and Creole, designed by the research collaborators. The confidentiality of the participants was rigorously preserved by ensuring the protection of personal information and avoiding any disclosure without the express consent of the participants. It is worth noting that this research is solely for academic and/or scientific purposes, and none of the participants will receive compensation and/or benefits for their participation.

## Results

The sociodemographic characteristics of the 345 participants are presented in Table 1, with a median age of 33 years. The majority were female (57%) and predominantly of Dominican nationality (95%). Most participants had at least a secondary education level, though 8% reported no formal education.

**Table 1:** Personal and couple risky sexual behavior (n = 345) This figure presents individual and partner-related risky sexual behaviors associated with STI transmission. The majority (96.5%, n = 333) reported prior sexual intercourse, with heterosexual contact being most common (95.4%, n = 329), followed by bisexual (2.3%, n = 8) and the same-sex contact (0.9%, n = 3). Regarding lifetime sexual partners, 52.8% (n = 182) had 1-2, 24.3% (n = 84) had ≥5, and 21.2% (n = 73) had 3-4. In the past year, 75.9% (n = 262) reported 1-2 partners, and 11.6% (n = 40) had ≥5. Genitovaginal sex was most common (91.6%, n = 316), followed by orogenital (36.8%, n = 127), masturbation (22.3%, n = 77), sex toy use (11.6%, n = 40), genitoanal (10.4%, n = 36), and nonpenetrative practices (9.6%, n = 30). Infidelity was experienced by 34% (n = 118), and 17% (n = 58) admitted being unfaithful. Condom use was inconsistent: 47% (n = 161) never used them, 35.1% (n = 121) used them occasionally, and 15.9% (n = 55) always. Alcohol consumption involved both partners in 34.8% (n = 120) of cases, neither in 33.9% (n = 117), only the participant in 17.4% (n = 60), and only the partner in 12.8% (n = 44). Illicit drug use was rare: 90.1% (n = 311) reported neither partner used, 4.6% (n = 16) both, 3.8% (n = 13) participant only, and 1.7% (n = 6) partner only STI: sexually transmitted infection

Sexual practice		Number of participants (%)
Have you had sex?	Yes	340 (98.3%)
	No	6 (1.7%)
With what sex?	Opposite sex	329 (95.4%)
	Same sex	3 (0.9%)
	Both sexes	8 (2.3%)
Number of sexual partners	1-2	182 (52.8%)
	3-4	73 (21.2%)
	≥5	84 (24.3%)
Number of sexual partners in the last year	1-2	262 (75.9%)
	3-4	19 (5.5%)
	≥5	40 (11.6%)
	None	19 (5.5%)
Type of sexual intercourse	Orogenital	127 (36.8%)
	Genitoanal	36 (10.4%)
	Genitovaginal	316 (91.6%)
	Nonpenetrative sex	33 (9.6%)
	Masturbation	77 (22.3%)
	Sex games	40 (11.6%)
History of infidelity	Personal	58 (16.88%)
	On the part of the couple	118 (34.2%)
Condom use during sexual intercourse	Never	161 (46.77%)
	Sometimes	121 (35.1%)
	Always	55 (15.9%)
Alcohol consumption	I	60 (17.4%)
	My partner	44 (12.8%)
	Both	120 (34.8%)
	None	117 (33.9%)
Illicit drug use	I	13 (3.8%)
	My partner	6 (1.7%)
	Both	16 (4.6%)
	None	311 (90.1%)

Risky sexual behaviors were highly prevalent (Table 2). A total of 96.5% reported having engaged in sexual intercourse, with heterosexual activity being the most common (95.4%). Bisexual and same-sex interactions were reported by 2.3% and 0.9% of participants, respectively. Regarding the number of lifetime sexual partners, 52.8% had one to two, 24.3% had five or more, and 21.2% had three to four. In the last year, 75.9% reported one to two sexual partners, and 11.6% had five or more.

**Table 2:** Sociodemographic characteristics of the participants (n = 346) The median age (X) was 33 years, with an IQR to indicate data dispersion. The predominant gender was female, accounting for 56.9% (n = 197), compared to 43.1% (n = 149) male participants. Regarding marital status, the largest group was those in a cohabitation, representing 40.2% (n = 139), followed by single individuals at 34.4% (n = 117), married individuals at 21.4% (n = 74), divorced at 2.6% (n = 9), and widowed at 1.4% (n = 5). In terms of nationality, the majority were Dominican, accounting for 94.8% (n = 328), while 5.2% (n = 18) were Haitian. As for educational level, 41.3% (n = 143) had completed secondary education, 28.9% (n = 100) had only completed primary education, 21.7% (n = 75) had university studies, and 8.1% (n = 28) had no formal education ^*^The age variable does not follow a normal distribution and is, therefore, presented as median ± IQR IQR: interquartile range

Variable	Number of participants (%)
Age^*^	33 ± 23
Sex	
Female	197 (57.9%)
Male	149 (43.1%)
Marital status	
Married	74 (21.4%)
Cohabitation	139 (40.2%)
Single	119 (34.4%)
Window/widower	5 (1.4%)
Divorced	9 (2.6%)
Nationality	
Dominican	328 (94.8%)
Haitian	18 (5.2%)
Educational level	
Elementary school	100 (28.9%)
High school	143 (41.3%)
University	75 (21.7%)
None	28 (8.1%)

In terms of sexual practices, 91.6% reported genitovaginal intercourse, followed by orogenital sex (36.8%), masturbation (22.3%), and sex toy use (11.6%). Less common practices included genitoanal intercourse (10.4%) and non-penetrative sexual activities (9.6%). Partner-related risk behaviors were also noted: 34% had experienced infidelity from their partner, and 17% admitted to being unfaithful. Condom use was inconsistent: 47% never used them, 35.1% used them occasionally, and only 15.9% reported always using condoms. Alcohol use was reported by both partners in 34.8% of cases, while illicit drug use was uncommon, with 90.1% indicating neither partner used such substances.

**Figure 1:**
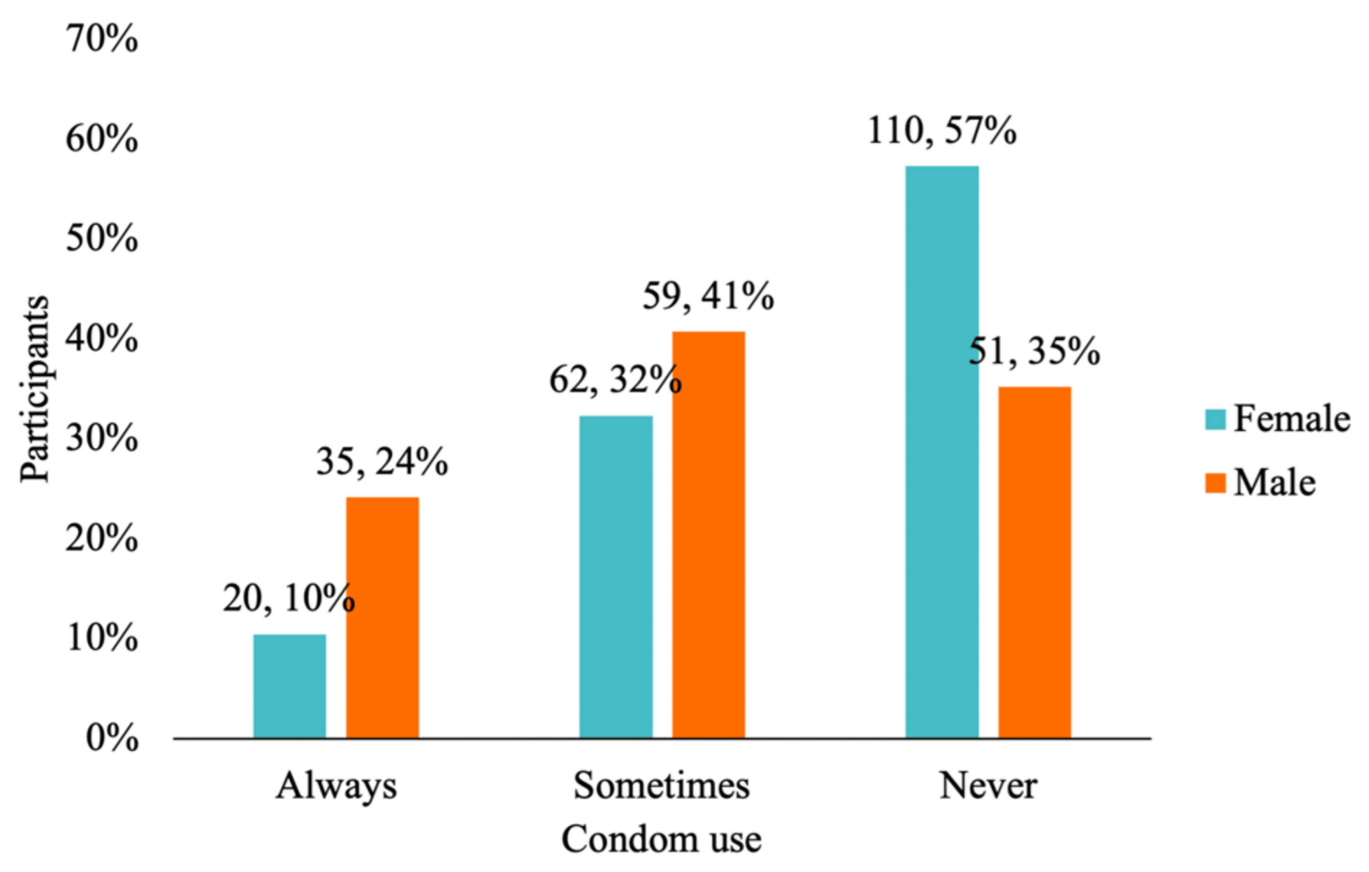
Frequency of male condom use by sex The distribution of condom use among male and female participants is shown. Among women, 57% (n = 110) reported never using condoms during sexual intercourse. In comparison, 35% of men (n = 51) reported the same behavior. Additionally, 41% of men (n = 59) stated that they use condoms occasionally, while 32% of women (n = 62) reported occasional use. Finally, 24% of men (n = 35) reported always using condoms, whereas only 10% of women (n = 20) reported consistent condom use

**Figure 2:**
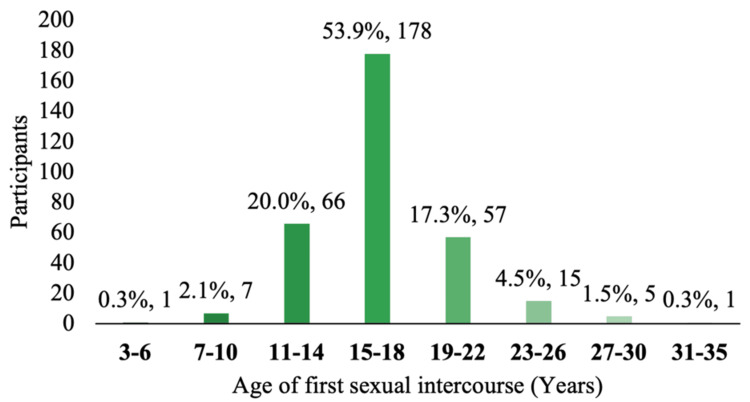
Age at first sexual intercourse The distribution of participants according to the age at which they had their first sexual intercourse is illustrated. The majority reported initiating sexual activity between the ages of 15 and 18 years, accounting for 53.9% (n = 178). Additionally, 20% (n = 66) had their first sexual experience between the ages of 11 and 14, and 17.3% (n = 57) between 19 and 22 years. The least frequent age ranges were 3-6 and 31-35 years, each reported by a single participant (0.3%)

**Figure 3:**
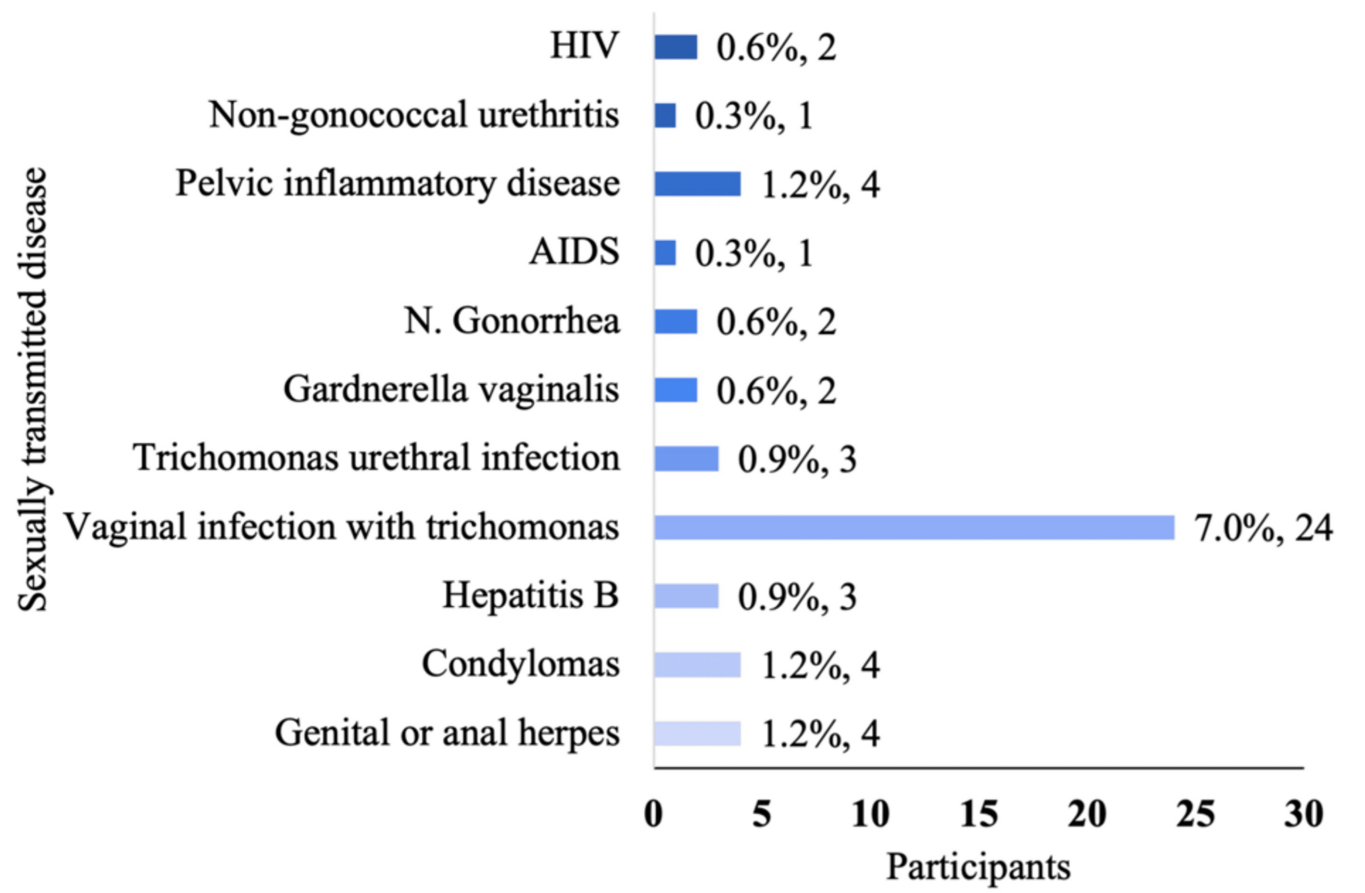
STIs in population studied The STIs that participants reported having had or currently have are presented. The most commonly mentioned STI was vaginal trichomoniasis, affecting 24 participants (7%). This was followed by pelvic inflammatory disease, genital warts (condylomas), and genital herpes, each reported by four participants (1.2%). Urethral trichomoniasis and hepatitis B were reported by three participants (0.9%). Gonorrhea, Gardnerella infection, and HIV were reported by two participants each (0.6%). The least frequently mentioned infections were AIDS and nongonococcal urethritis, each affecting one participant (0.3%) STIs: sexually transmitted infections

**Figure 4:**
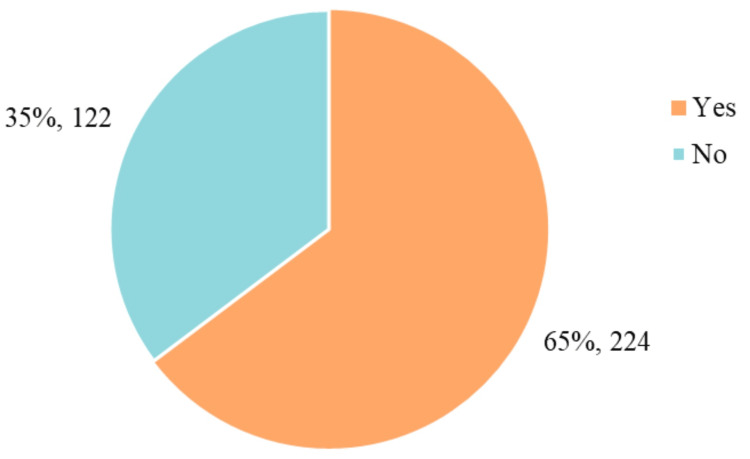
Sexual education (n = 346) The participants' history of receiving sexual education is illustrated. According to the results, 65% (n = 224) of respondents reported having received sexual education. In contrast, 35% (n = 122) stated they had not received any educational experience in this area

Figure 1 illustrates that the most common age range for first sexual intercourse was 15-18 years (53.9%), followed by 11-14 years (20%) and 19-22 years (17.3%). Figure 2 details the prevalence of STIs in the sample, with vaginal trichomoniasis being the most frequently reported (7%). Other infections, including pelvic inflammatory disease, genital herpes, condyloma, gonorrhea, and HIV, were reported by fewer than 2% of participants. Figure 3 shows that 64% of participants had received sexual education, primarily through school or healthcare providers. However, as shown in Figure 4, condom use was significantly lower among women, with 57% reporting never using condoms during sexual activity, compared to 35% of men. Only 10% of women reported always using condoms, vs. 24% of men. These findings highlight the widespread prevalence of high-risk sexual behaviors and limited consistent condom use, despite a majority reporting prior exposure to sexual education.

## Discussion

In this study, the risk factors related to sexual practices and the transmission of STIs were evaluated based on the most common age range for first sexual experience, the most frequent STIs within the population, the frequency of condom use, and the types of high-risk sexual behaviors among residents over the age of 18 in the Santa Rosa sector of Baní.

The data collected reveal significant risk behaviors associated with sexual activity in the study population. Most participants reported having engaged in sexual intercourse, with 161 indicating they never used condoms, and 121 reporting occasional use. Additionally, 118 participants had experienced infidelity from their partners, while 58 admitted to having sexual relations with someone other than their current partner. Regarding the number of sexual partners in the last year, 40 individuals reported having five or more, and at least 19 reported having three to four. This information is reflected in Table 2. These findings align with a study conducted in Venezuela by Narváez-Jaramillo et al. [9], in which 84% of respondents stated they did not use condoms during sexual intercourse. This highlights the ongoing relevance of risky sexual behaviors and the pressing need for educational and preventive interventions tailored to the population studied.

In terms of the age of first sexual intercourse, it is noteworthy that 174 individuals reported initiating sexual activity between the ages of 13 and 17, making this the most represented age group in the sample. Furthermore, 102 individuals began sexual activity between the ages of 18 and 22, representing a significant portion of early adulthood sexual initiation. Less frequently, participants reported their first sexual experience between the ages of three and seven, and 33 to 35, with two and one individuals, respectively (see Figure 2). These results are consistent with a study conducted by Medina et al. in Argentina, which found that 59% of participants had their first sexual experience before the age of 14 [10], with ages ranging from 13 to 27 years. Despite differences in geographic and social context, there appears to be a pattern in early sexual initiation across various regions. Scientific literature indicates that earlier sexual initiation can result in adverse psychological, social, and economic consequences. Adolescents who begin sexual activity early are more likely to engage in high-risk sexual behavior, such as having multiple sexual partners (either consecutively or concurrently) or inconsistent use of protection. These behaviors increase their risk for unplanned pregnancies and acquiring STIs [10]. Countries such as Chile have reported associations between inadequate sexual education and earlier initiation of sexual activity and inconsistent contraceptive use among adolescents [11].

In terms of condom use by sex, a striking 110 women reported never using condoms during sexual intercourse, while 51 men gave the same response. According to a study conducted in Colombia by Arrivillaga et al., only 17.4% of participants used a condom during their last sexual encounter. When broken down by gender, 22.1% of men reported using a condom compared to 13.7% of women, indicating a statistically significant difference [12]. These findings reveal disparities in condom use behaviors and emphasize the need for tailored intervention strategies.

When analyzing the STIs recalled by participants, vaginal trichomoniasis emerged as the most commonly reported infection, affecting 24 individuals (7% of the sample) (see Figure 3). This finding aligns with a study by Ambrozio et al. in Brazil, where 27 out of 300 participants (9%) tested positive for trichomoniasis [13], suggesting a comparable prevalence across regions. Other infections reported include pelvic inflammatory disease, genital warts, and herpes, each affecting four participants. Less commonly, two participants reported gonorrhea, Gardnerella, or HIV, while AIDS and nongonococcal urethritis were reported by one participant each (see Figure 3).

Regarding sexual education, 221 participants confirmed having received some form of sexual education, while 121 stated they had not (see Figure 4). These findings offer valuable insights into the population's sexual health practices and knowledge, supporting the need for targeted interventions.

One significant limitation of this study was the availability of participants during the scheduled data collection period. Despite efforts to coordinate visits conveniently, external factors such as work, family responsibilities, or other commitments may have limited participation.

Another key limitation was the time constraints during the data collection phase. The study faced a restricted time frame that affected both the depth and breadth of information gathered. These time constraints impacted the study design and limited the ability to reach a broader, more diverse sample or conduct more detailed follow-ups on specific aspects.

## Conclusions

The findings of this study emphasize the urgent need for comprehensive sex education programs focused on reducing high-risk behaviors such as inconsistent condom use, early sexual initiation, and infidelity. The high prevalence of STIs, particularly vaginal trichomoniasis, highlights gaps in prevention and access to care. Targeted educational interventions could significantly improve sexual health outcomes in the studied population.

## Appendices

Data collection tool

1. Survey no. _____

Section I: Sociodemographic Data

2. Age _____ years 3. Sex: Male ( ) Female ( ) 4. Nationality: Dominican ( ) Haitian ( ) Other ( ) 5. Marital status: Married ( ) Common-law union ( ) Single ( ) Widowed ( ) Divorced ( ) 6. Educational level:

Primary

Secondary (high school)

University

None

Section II: Personal and Partner Behavioral Data

7. Have you had sexual intercourse? No ( ) Yes ( )

8. With what sex? Opposite sex ( ) Same sex ( ) Both sexes ( )

9. At what age did you have your first sexual experience? _____

10. How many sexual partners have you had? a) 1-2 b) 3-4 c) ≥5

11. How many people have you had sexual relations with in the past year? a) 1-2 b) 3-4 c) ≥5

12. Your sexual relationships are: (You may select more than one) Orogenital (oral sex) ( ) Genitoanal (anal sex) ( ) Genitovaginal (vaginal penetration) ( ) Sexual activity without penetration ( ) Masturbation ( ) Sexual games ( )

13. Have you ever had a sexually transmitted infection (STI)? No ( ) Yes ( )

14. If yes, did you receive treatment? No ( ) Yes ( )

15. Do you have intimate relationships with people other than your current partner? Yes ( ) No ( )

16. Condom use in your sexual relations: Always ( ) Sometimes ( ) Never ( )

17. If you use condoms, with whom or when do you use them? Regular or steady partner ( ) New partners ( )

18. Do you or your partner consume alcohol? I do ( ) My partner does ( ) Both ( ) Neither ( )

19. Do you or your partner use drugs? I do ( ) My partner does ( ) Both ( ) Neither ( )

20. Have you ever experienced infidelity by your partner? Yes ( ) No ( )

Section III: Personal and Current Partner’s Medical History

21. Have you ever had, or do you currently have, any of the following conditions?

Genital or anal herpes( )( )

Genital warts (condylomas)( )( )

Hepatitis B( )( )

Vaginal trichomonas infection( )( )

Urethral trichomonas infection( )( )

Gardnerella( )( )

Gonorrhea( )( )

Syphilis( )( )

AIDS( )( )

Pelvic inflammatory disease( )( )

Nongonococcal urethritis( )( )

HIV( )( )

I have had none of these( )( )

I don’t know( )

I’m sure I was diagnosed, but don’t remember the name of the disease( )

22. Has your sexual partner ever had or does he/she currently have any of the above-mentioned conditions? Has not had any ( ) Other ( ) What? _______ I don’t know ( )

Section IV: Sexual Education

23. Have you received sexual education? Yes ( ) No ( )

24. Through what means did you receive it? School ( ) Parents ( ) Friends ( ) Doctor ( ) Television ( ) Other ( )

25. How would you rate the sexual education you have received regarding these topics? Poor or none ( ) Rare or average ( ) Systematic or good ( )
